# The Challenges in Investigating and Managing Dyspnoea in a Young Athlete With Asthma and Sickle Cell Trait

**DOI:** 10.1002/rcr2.70796

**Published:** 2026-11-08

**Authors:** Nicole Niu, Eskandarain Shafuddin

**Affiliations:** ^1^ Department of Respiratory Medicine Waikato Hospital Hamilton New Zealand

**Keywords:** asthma, CPET, sickle cell trait

## Abstract

Asthma and sickle cell trait (SCT) are common conditions in adolescents and do not usually preclude high‐level athletic participation. Both may be associated with exertional symptoms, creating diagnostic uncertainty and management challenges in athletes. We report the case of a 17‐year‐old football player with asthma and SCT who presented with exertional dyspnoea and muscle cramps. Routine investigations, including lung function tests, chest imaging and echocardiography, were within normal limits. Cardiopulmonary exercise testing (CPET) demonstrated exercise‐induced dynamic hyperinflation, consistent with active asthma physiology during exercise. However, preserved peak oxygen uptake, anaerobic threshold and breathing reserve indicated this was not severe enough to be the predominant driver of his exercise limitation. Exercise was ultimately terminated volitionally due to leg fatigue, rather than significant ventilatory, cardiac or metabolic limitations, allowing reassurance and targeted management.

## Graphical abstract

We present the case of a competitive adolescent athlete with asthma and sickle cell trait whose exertional symptoms despite inhaler treatment posed a diagnostic challenge. Although exercise‐induced asthma physiology was detected on cardiopulmonary exercise testing, his overall exercise capacity was appropriate for a highly trained athlete, with no significant cardiopulmonary limitation.

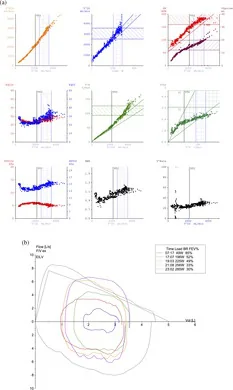

## 1 Introduction

Exercise intolerance or dyspnoea in adolescent athletes is frequently attributed to asthma or exercise‐induced bronchoconstriction (EIB). However, coexisting chronic conditions such as SCT can complicate assessment and management. While usually benign, SCT can rarely cause severe complications during high‐intensity exertion.

Conventional investigations such as pulmonary function tests, echocardiography and imaging may be insufficient to identify the underlying physiological driver of exertional dyspnoea. CPET offers an integrated evaluation of cardiovascular, pulmonary and metabolic responses to exercise, and is increasingly recognised as the gold standard for investigating unexplained dyspnoea and exercise limitation.

We present the case of a competitive adolescent athlete with asthma and SCT whose exertional symptoms despite inhaler treatment posed a diagnostic challenge. Although exercise‐induced asthma physiology was detected on CPET, his overall exercise capacity was appropriate for a highly trained athlete, with no significant cardiopulmonary limitation.

## 2 Case Report

A 17‐year‐old competitive football player was referred for breathlessness, fatigue, left‐sided chest discomfort and bilateral calf muscle cramps during high‐intensity training and competition. He is a never smoker with childhood asthma managed with inhaled bronchodilators (combined budesonide/formoterol), and a leukotriene antagonist, as well as SCT manifesting as microcytosis without anaemia. His medical history also included hay fever, eczema and possible Raynaud's phenomenon.

Initial workup included serial normal chest radiographs and a normal ventilation‐perfusion scan. Post‐bronchodilator spirometry and diffusion capacity were within normal limits, with FEV1 4.51 L (104% predicted), FVC 4.78 L (94% predicted) and DLCO 98%.

In view of the patient's persistent breathlessness during intensive training despite inhaler treatment, CPET was performed (Figure 1a) on a cycle ergometer using a continuous 15 W/min ramp to volitional termination. Data were analysed using SentrySuite software, with predicted values derived from the Wasserman et al. reference values [1]. This demonstrated peak VO_2_ 64.3 mL/min/kg (134% predicted), anaerobic threshold (AT) at 69% predicted VO_2_ max, VE/VCO_2_ slope at AT 23, oxygen pulse response 24.6 mL (154% predicted), dynamic hyperinflation (Figure 1b) and adequate breathing reserve at end exercise (29%). Peak workload was 282 W (115% predicted). Oxygen saturations, heart rate and blood pressure responses remained normal throughout exercise. Although a left ventricular hypertrophy pattern was noted on ECG, a transthoracic echocardiogram did not reveal ventricular hypertrophy. Exercise was terminated by leg fatigue rather than his presenting symptoms of chest discomfort or muscle cramps, though he did report a cough affecting his breathing near peak exercise. These findings suggested physiological evidence of asthma without significant respiratory limitation, and with no evidence of cardiac or metabolic exercise limitation (Table 1).

**FIGURE 1:**
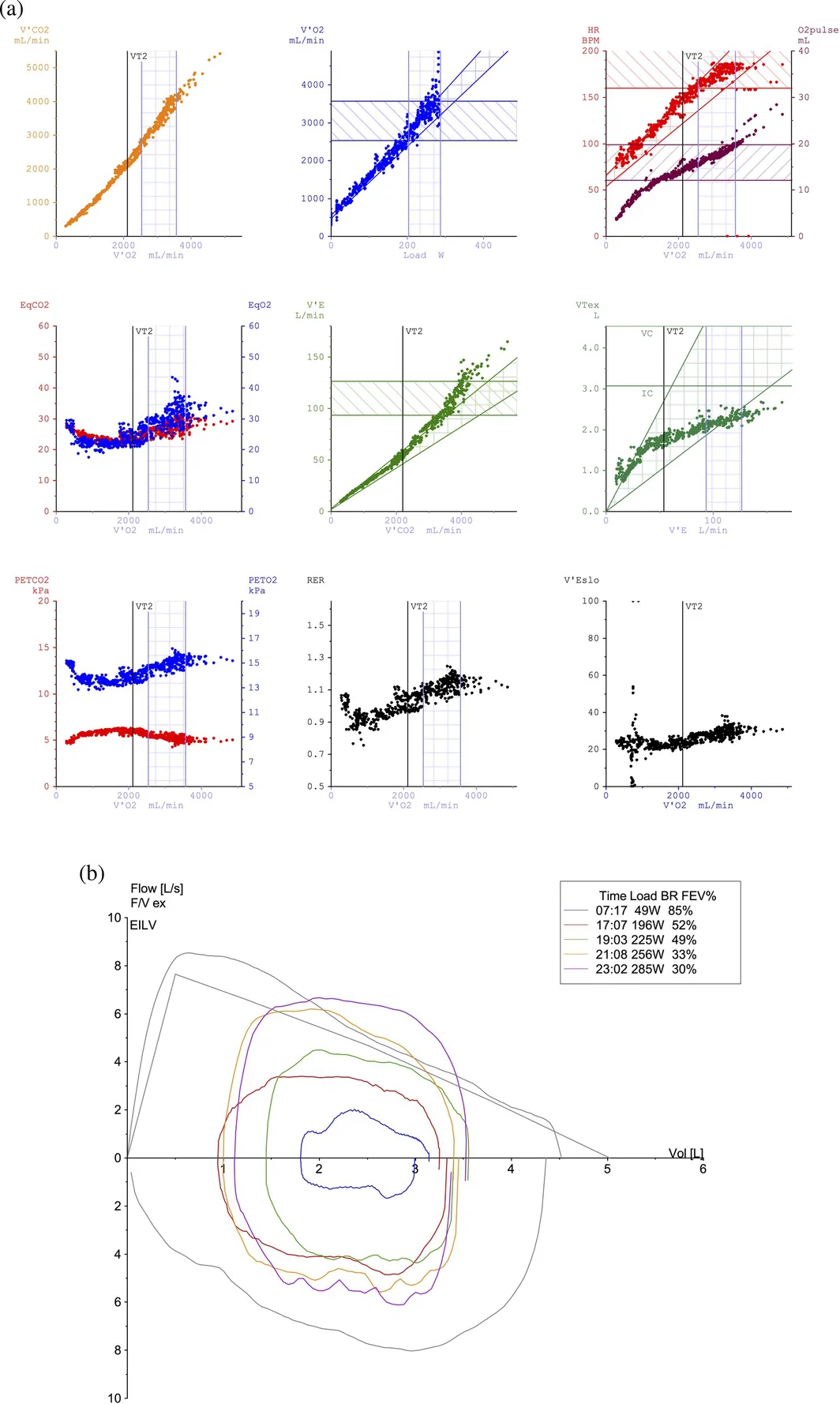
Cardiopulmonary exercise testing findings. (a) Nine‐panel CPET plot demonstrating preserved aerobic capacity with peak VO_2_ of 64.3 mL/kg/min, normal ventilatory efficiency (VE/VCO_2_ 23), preserved oxygen pulse response and adequate breathing reserve. AT occurred at 69% predicted VO_2_ max. (b) Exercise flow‐volume loops during CPET demonstrating progressive dynamic hyperinflation during increasing exercise intensity, suggestive of exercise‐induced asthma physiology.

**TABLE 1:** CPET results and predicted values.

CPET parameters	Result	Predicted/reference range	% Predicted
Peak VO_2_ max	64.3 mL/min/kg	48.0 mL/min/kg	134%
Anaerobic threshold	2110 mL/min (33.2 mL/min/kg)	40%–80% of predicted VO_2_ max [2]	69%
VE/VCO_2_ slope at AT	23	< 34 [2]	
Oxygen pulse response	24.6 mL	15.9 mL	154%
Breathing reserve	29%	> 15% [2]	
Peak workload	282 W	246 W	115%

He reported greater symptomatic relief with ipratropium compared to combined budesonide/formoterol, and was advised to continue inhaled bronchodilator therapy alongside montelukast and loratadine. He was counselled on adequate hydration during intensive exercise to reduce the risk of SCT‐related complications, and advised to seek urgent review if experiencing persistent chest pain, palpitations, musculoskeletal pain or left upper quadrant abdominal pain suggestive of splenic infarction. Surveillance included 3‐monthly blood tests and a repeat echocardiogram at 1 year to monitor for ventricular hypertrophy.

## 3 Discussion

This case highlights the multifactorial nature of impaired exercise tolerance and the importance of a structured, multi‐modal approach when evaluating exertional symptoms in patients with overlapping chronic conditions. Despite normal resting investigations, he reported exertional fatigue, muscle cramps and breathlessness, raising concerns regarding asthma severity, potential SCT‐related complications or occult cardiac dysfunction. The aetiology of his muscle cramps was not clarified by CPET. Possible contributors include dehydration or heat‐related cramping, benign exercise‐associated cramps, or a contribution from his SCT, though this is less likely given the reassuring CPET metabolic profile. Notably, his presenting symptoms of muscle cramps and chest discomfort were not reproduced during CPET, which was terminated because of leg fatigue. This is a limitation when interpreting how fully the CPET findings explain his overall symptoms.

Dynamic hyperinflation on CPET pointed to asthma as a contributing factor despite normal baseline spirometry, highlighting the importance of exercise‐based testing to capture subtle functional limitation. It should be noted that CPET is not a validated diagnostic test for EIB. The dynamic hyperinflation observed here is suggestive rather than diagnostic of an asthma‐related ventilatory limitation, and dedicated protocols such as eucapnic voluntary hyperventilation are required for formal EIB diagnosis [3]. Bronchodilator responsiveness and bronchoprovocation testing were not performed, as it was not considered safe to withhold his preventer inhaler given his need for ongoing asthma treatment while actively in training. Fractional exhaled nitric oxide (FeNO), measured 1 year prior to CPET, was within the intermediate range at 31 ppb. In highly trained athletes, peak VO_2_ reflects overall aerobic potential, but AT is a better predictor of sustained exercise performance than peak VO_2_ [4]. Although his peak VO_2_ of 134% predicted reflects superior aerobic capacity, it could be argued that the AT reached is ‘early’ for his level of athleticism. Wasserman‐derived prediction equations may underestimate peak VO_2_ in young, highly trained endurance athletes [5]. Therefore, while his AT falls within the accepted normal range, this should be interpreted in the context of his high aerobic capacity, which may itself be underestimated by the reference equation.

While this patient had sickle cell trait rather than sickle cell disease, data describing CPET findings in sickling disorders are limited. Table 2 provides an overview of expected exercise physiology patterns in asthma and sickle cell disease, acknowledging that individuals with SCT are generally expected to demonstrate milder or absent physiological impairment.

**TABLE 2:** Expected CPET findings in patients with asthma and sickle cell disease.

CPET parameters	Asthma	Sickle cell disease
Typical exercise limitation pattern	Primarily ventilatory	Mixed cardiovascular, ventilatory, metabolic
Peak VO_2_	Usually normal. May be reduced in severe airflow limitation or deconditioning	Often reduced due to chronic anaemia, impaired oxygen delivery and decreased aerobic capacity
Anaerobic threshold	Usually normal. May be reduced/early in severe airflow limitation or deconditioning	Often reduced/early, reflecting early lactic acidosis, due to impaired muscle oxygen extraction and reduced aerobic capacity
Breathing reserve	May be reduced in severe asthma due to airflow obstruction and dynamic hyperinflation	Usually preserved
Dynamic hyperinflation	May occur during exercise due to expiratory airflow limitation	Not a characteristic feature
VE/VCO_2_	Usually normal. May be elevated in severe asthma or dysfunctional breathing	May be elevated, particularly with pulmonary vascular dysfunction or cardiovascular disease
O_2_ saturation	Usually normal. May be reduced in severe asthma due to exercise induced bronchoconstriction	May be reduced with significant anaemia, pulmonary vascular disease or cardiovascular disease
Exercise limiting symptom	Dyspnoea, chest tightness, wheeze	Fatigue, dyspnoea, pain, muscular symptoms

An LVH pattern on ECG without echocardiographic structural abnormalities is considered a normal physiological response in athletes, further reassuring against pathological hypertrophy as a cause of his symptoms.

Individuals with SCT typically have a normal life expectancy without sickle cell crises, though extreme exertion with relative hypovolaemia, hypoxia and acidosis can cause erythrocyte sickling and microvascular occlusion, with rare reported cases of exercise‐induced rhabdomyolysis, exertional collapse associated with sickle cell trait (ECAST) and splenic infarctions at high altitude [6]. A recent systematic review found no evidence linking SCT alone to sudden death in the absence of exertion‐related rhabdomyolysis or acute vaso‐occlusive crises, emphasising that such fatal outcomes reflect severe metabolic complications rather than the trait alone [7].

More invasive approaches to CPET may provide additional physiological information in selected cases where non‐invasive findings are inconclusive. Serial arterial or capillary lactate measurement allows direct determination of the lactate threshold, which closely parallels the ventilatory‐derived AT used here. Arterial blood gas sampling at rest and at peak exercise also allows calculation of the alveolar‐arterial (A‐a) oxygen gradient, which can provide additional information on ventilation‐perfusion mismatch or diffusion limitation. Such methods can be valuable adjuncts when differentiating heart failure with preserved ejection fraction (HFpEF) from noncardiac causes of dyspnoea [8]. Where non‐invasive testing remains ambiguous, invasive CPET (iCPET) using right heart catheterisation is considered the gold standard [9]. It allows direct measurement of right atrial, pulmonary arterial and capillary wedge pressures alongside cardiac output during exercise. This can identify exercise‐induced pulmonary hypertension, HFpEF and other causes of dyspnoea not clearly defined by non‐invasive methods, such as mitochondrial myopathies or preload‐dependent limitations.

In this young athlete with asthma and SCT, CPET was useful in clarifying the physiological contributors of his exercise intolerance. It identified exercise‐related asthma physiology without respiratory limitation, while excluding significant cardiac or metabolic limitations, enabling tailored management and safe participation in competitive sport.

## Author Contributions

**Nicole Niu:** writing – original draft, writing – review and editing. **Eskandarain Shafuddin:** supervision, writing – review and editing.

## Consent

The authors declare that written informed consent was obtained from the patient and his parent for the publication of this manuscript and accompanying images using the consent form provided by the Journal.

## Conflicts of Interest

The authors declare no conflicts of interest.

## Data Availability

Data sharing not applicable to this article as no datasets were generated or analysed during the current study.
